# Early childhood caries and type 1 diabetes mellitus—Evidence on primary teeth and oral health: A systematic review

**DOI:** 10.17305/bb.2026.13488

**Published:** 2026-02-11

**Authors:** Jelena Komšić, Ljiljana Andrijević, Mirjana Hadnađev, Bojan Petrović, Duška Blagojević, Isidora Nešković, Sanja Vujkov

**Affiliations:** 1 Faculty of Medicine, University of Novi Sad, Novi Sad, Serbia; 2 Institute for Pulmonary Diseases of Vojvodina, Sremska Kamenica, Serbia

**Keywords:** Child, diabetes mellitus type 1, dental caries, primary tooth, oral health, saliva, periodontal diseases, preventive dentistry.

## Abstract

Early childhood caries (ECC) among children with type 1 diabetes mellitus (T1D) remains poorly characterized in the primary dentition (≤71 months), despite frequent reports of salivary and periodontal alterations that could plausibly increase caries susceptibility. This systematic review aimed to compare ECC prevalence and severity in children with T1D versus healthy controls and to examine associations with glycemic control, salivary parameters, periodontal indices, and preventive-care behaviors. Following Preferred Reporting Items for Systematic Reviews and Meta-Analyses (PRISMA) 2020 and International Prospective Register of Systematic Reviews (PROSPERO) registration (CRD42024602599), we searched five databases and Google Scholar for eligible observational studies; due to substantial heterogeneity in age ranges, dentition stages, and outcome reporting, findings were synthesized narratively. Twelve studies met inclusion criteria. Evidence on caries experience in primary teeth was inconsistent, with some studies reporting higher dmft in T1D and others showing comparable or lower values than controls. In contrast, secondary outcomes showed more coherent patterns: T1D was frequently associated with reduced salivary flow, diminished buffering capacity, lower salivary pH, and early gingival inflammation, while poorer glycemic control was commonly linked to worse oral-health parameters; preventive dental attendance and fluoride-related practices were variably reported and often irregular. Overall, current evidence does not conclusively demonstrate a higher ECC burden in T1D, but consistent salivary and periodontal vulnerability supports early, integrated preventive oral-health care within pediatric diabetes management.

## Introduction

Type 1 diabetes mellitus (T1D) is a chronic autoimmune disorder that primarily affects children and is characterized by absolute insulin deficiency and long-term metabolic instability [1–3]. While systemic complications are well documented, oral health, particularly in preschool-aged children, remains an underexplored yet clinically significant aspect of T1D [4–7]. Chronic hyperglycemia can disrupt salivary gland function, increase inflammatory activity, and potentially elevate the risk of oral diseases in young children [8–10]. Early childhood caries (ECC), defined as the presence of one or more decayed, missing, and filled teeth (dmft) in children aged 71 months or younger, is among the most prevalent pediatric conditions and carries lifelong consequences [11, 12]. Numerous observational studies have evaluated dental caries and oral health parameters in children with T1D, but the findings are inconsistent. Some studies report increased caries severity, while others indicate similar or even lower dmft levels in T1D children compared to their healthy peers, possibly due to stricter dietary supervision or more frequent medical monitoring [13–18]. Complicating interpretation, many studies encompass broad age ranges that do not align with the ECC definition, mix outcomes from primary and permanent dentition, or lack stratified data for children aged 71 months or younger [15]. Reduced salivary flow, diminished buffering capacity, and early gingival inflammation may heighten susceptibility to ECC, despite current evidence showing inconsistent dmft patterns. Given these uncertainties, a focused review confined to the ECC age window is essential. This review aims to determine whether children aged 71 months or younger with T1D exhibit a higher burden of ECC compared to their non-diabetic peers, summarize findings related to salivary and periodontal parameters in this population, and evaluate preventive care engagement pertinent to early caries risk.

The primary objective of this systematic review was to assess whether preschool-aged children with T1D (≤71 months) have a higher burden of ECC in the primary dentition compared to healthy children.

Secondary objectives included examining existing evidence regarding salivary function (unstimulated/stimulated flow rate, buffering capacity, pH level), gingival and periodontal status (gingival index [GI], plaque index [PI], bleeding on probing [BOP]), glycemic control (glycated hemoglobin [HbA1c]) and its association with oral outcomes, and preventive oral health behaviors, including dental attendance, fluoride use, and home care practices.

## Materials and methods

### Protocol and registration

This systematic review was conducted in accordance with the Preferred Reporting Items for Systematic Reviews (PRISMA) 2020 guidelines. The review protocol was prospectively registered in the International Prospective Register of Systematic Reviews (PROSPERO) under the registration number CRD42024602599. The protocol outlined the review objectives, eligibility criteria, search strategy, data extraction methods, and risk of bias assessment, as well as the planned approach for narrative data presentation and interpretation. No amendments were made to the registered protocol.

### Eligibility criteria

Studies were eligible for inclusion if they examined children with T1D and reported outcomes related to the primary dentition. ECC, defined as caries occurring in children aged 71 months or younger, was the primary outcome of interest. Studies that included broader pediatric age ranges were retained only if they provided extractable primary dentition outcomes or relevant secondary oral health parameters. These studies were not used to infer ECC prevalence directly but were included to inform biological plausibility and oral health patterns relevant to primary teeth within the ECC framework. Included studies were required to feature a healthy non-diabetic comparison group and to report ECC outcomes using dmft/decayed, missing, and filled surfaces (dmfs) or International Caries Detection and Assessment System (ICDAS) criteria. Secondary outcomes of interest included salivary function (flow rate, buffering capacity, pH), periodontal indices (GI, PI, BOP), mucosal findings, and preventive oral health behaviors. Only observational human studies (cross-sectional, case–control, or cohort) were eligible. Reviews, case reports, editorials, and studies lacking primary dentition data or a suitable comparison group were excluded. For feasibility, the search was limited to English-language publications, with acknowledgment of potential language bias.

**Figure 1.**
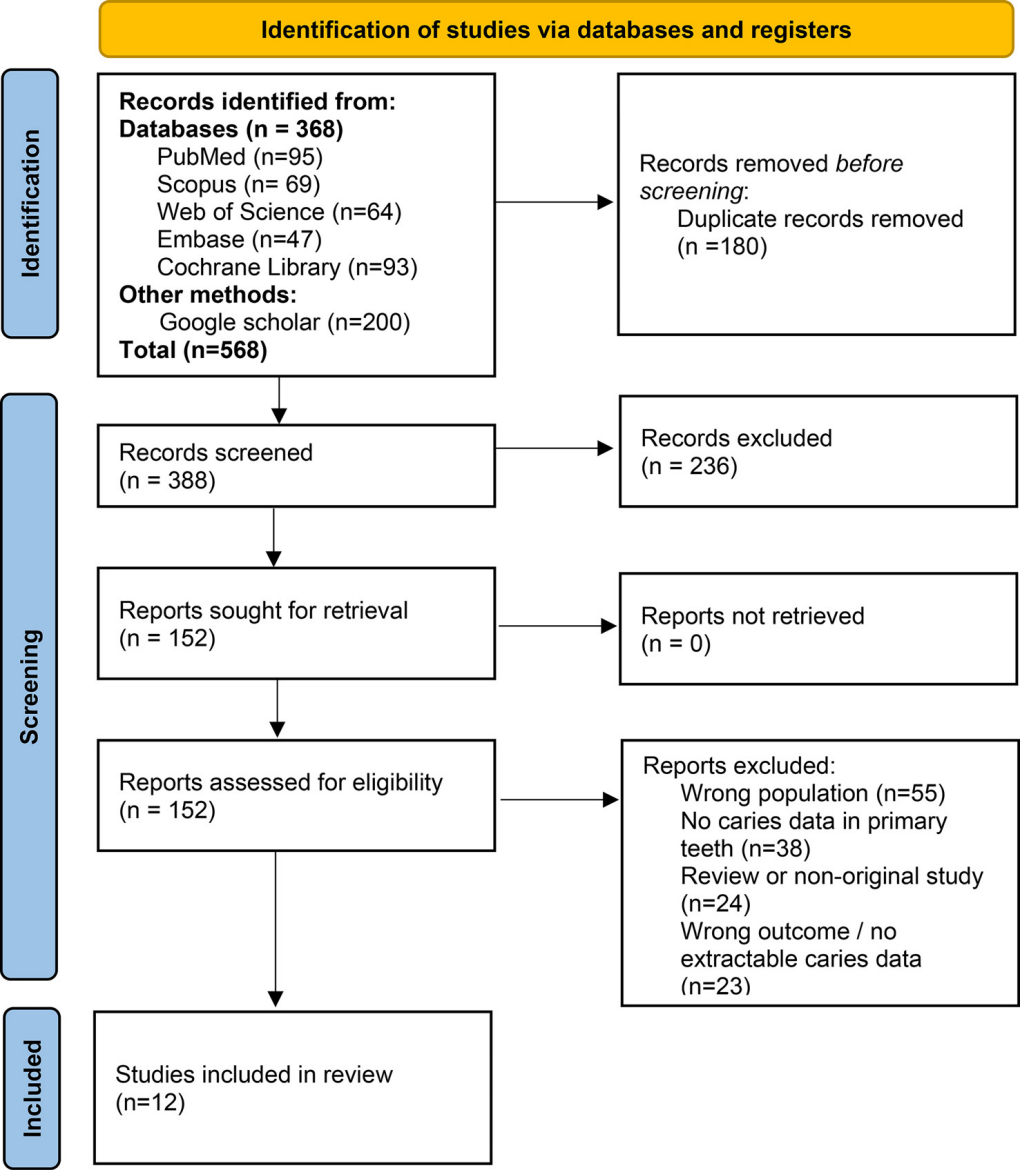
PRISMA flow diagram.

### Information sources

A systematic search was performed in PubMed (National Center for Biotechnology Information, NCBI), Scopus (Elsevier), Web of Science Core Collection (Clarivate), Embase (Elsevier), and Cochrane CENTRAL (Cochrane Library), in accordance with PRISMA 2020 guidelines. The search was conducted in April–May 2025 without date restrictions and was limited to English-language publications. Google Scholar was used as a supplementary source for citation screening, limited to the first 200 records sorted by relevance. Reference lists of included studies and relevant systematic reviews were also manually screened. Full database-specific search strategies are provided in Table S1.

### Search strategy

A tailored search strategy was developed for each database using free-text keywords, iteratively refined to maximize sensitivity. Boolean operators (AND/OR) and truncation were applied as appropriate. The search was structured around three core domains: population (children), exposure (T1D), and outcomes related to dental caries (ECC, dmft, primary teeth). Full, database-specific search strings are provided in Supplementary Material S1.

### Study selection

All records identified through database searches and other sources were screened for duplicates, which were removed through manual cross-checking. Titles and abstracts were independently screened by two reviewers (JK, BP). Full-text articles were retrieved for potentially eligible studies and assessed independently by the same reviewers. Disagreements were resolved through discussion, with consultation of a third reviewer (IN) when necessary. The study selection process is summarized in the PRISMA 2020 flow diagram.

### Data collection process

A standardized extraction form was utilized. Two reviewers (JK, BP) independently extracted study characteristics and outcome data. Discrepancies were resolved through discussion, with consultation of a third reviewer (IN) when required. Permanent dentition outcomes, assessed using the decayed, missing, and filled teeth (DMFT) index, were extracted for contextual purposes but were not included in the primary emphasis.

Data extracted from each study included identification, design, and population characteristics. The primary outcomes included measures of caries in the primary dentition, specifically caries prevalence, the dmft index, the decayed, extracted, and filled teeth (deft) index, and the classification of ECC and severe early childhood caries (S-ECC). When reported, secondary outcomes included salivary parameters, glycemic control indicators (HbA1c), periodontal indices, additional oral health findings, and preventive care behaviors. These data informed the narrative synthesis. Narrative synthesis of included studies are presented in Table 1.

### Risk of bias evaluation

Methodological quality was assessed using the Joanna Briggs Institute (JBI) Critical Appraisal Checklists for observational studies. The JBI appraisal items are listed in Table 2, and study-level risk-of-bias assessments are presented in Table 3. Two reviewers independently evaluated each study, with disagreements resolved through discussion or consultation of a third reviewer. Studies were categorized as having low, moderate, or high risk of bias. Due to the narrative nature of the synthesis and the limited number of studies for each outcome, formal assessments of reporting bias (e.g., funnel plots or Egger’s test) and evidence certainty (e.g., Grading of Recommendations Assessment, Development and Evaluation (GRADE)) were not conducted. Instead, potential selective reporting was qualitatively evaluated.

### Synthesis of results

A narrative synthesis was undertaken due to substantial heterogeneity across studies in age ranges, outcome definitions, and reporting. Too few studies provided extractable primary dentition data specific to children aged 71 months or younger to permit meaningful meta-analysis. Findings are therefore presented descriptively and organized by major outcome domains, including caries experience, salivary function, periodontal status, glycemic control, and preventive behaviors. Outcomes were extracted as reported, without data conversion or imputation.

All data management and descriptive tabulation were performed using Microsoft Excel (Version 2304). Extracted variables were organized into standardized tables to support consistent cross-study comparison. EndNote 21 was used for reference management. A qualitative, narrative synthesis approach was applied due to substantial methodological heterogeneity, including variation in study designs, age ranges, dentition stages, diagnostic criteria, and inconsistently reported dmft data, which precluded meaningful quantitative pooling. Consequently, no meta-analytical or statistical modeling procedures were undertaken.

### Ethics and dissemination

This study is a systematic review of previously published literature and did not involve the collection of new data from human participants; therefore, formal ethical approval was not required. All included studies were independently published and are assumed to have received appropriate ethical approvals from their respective institutions.

## Results

### Study selection

The search identified a total of 568 records (368 from databases and 200 from Google Scholar). After manually removing duplicates, titles and abstracts were screened, followed by a full-text assessment of potentially eligible studies. Twelve studies met the inclusion criteria for qualitative synthesis. A PRISMA 2020 flow diagram summarizing the selection process is presented in Figure 1.

### Study characteristics

The 12 included studies were conducted across Europe, Asia, the Middle East, and South America. All studies employed observational designs (cross-sectional or case-control), with sample sizes ranging from 46 to 264 participants. Most studies assessed caries outcomes in primary or mixed dentition and also examined salivary characteristics (flow rate, buffering capacity, pH), periodontal indices (GI, PI, BOP), and/or glycemic control (HbA1c levels). While some studies reported outcomes specifically for children aged ≤71 months, others included broader age ranges; nonetheless, all studies were retained in the narrative synthesis due to their relevance in elucidating the biological mechanisms linking T1D and oral health. Table 1 provides an overview of the key characteristics of all included studies. Given the variability in age ranges, dentition reporting, and methodological approaches, all findings are presented narratively.

**Table 1:** Narrative synthesis of included studies (*n* ═ 12)

**First author (year)**	**Country**	**Sample description**	**Study design**	**Comparison group**	**Outcome measures**	**Observed association**	**Key findings**	**Relevance to ECC**
Blumer (2018)	Israel	Children ≤6 yrs, T1D vs controls	Cross-sectional	Yes	ECC, salivary pH	ECC ↑; pH ↓	Lower pH, higher ECC in T1D	Direct ECC relevance
Manjushree (2022)	India	50 T1D, 50 controls, 6–12 yrs	Cross-sectional	Yes	DMFT/deft	DMFT/deft ↑; saliva ↓	Higher caries, lower saliva	Salivary/caries relevance
Sheikh (2017)	Kenya	82 children, 3–18 yrs	Cross-sectional	Yes	DMFT/dmft	DMFT/dmft ↑; gingivitis ↑	Higher caries, gingivitis	Metabolic–oral link
Dubey (2018)	India	30 T1D, 60 controls, 5–14 yrs	Comparative	Yes	DMFT/deft, saliva	DMFT/deft ↑; saliva altered	Higher caries, altered saliva	Systemic salivary effects
Lai (2017)	Italy	98 T1D, 203 controls, 3–14 yrs	Casecontrol	Yes	dmft	dmft ↑*	Higher dmft; OR 1.9	Primary dentition relevance
Salehi (2024)	Iran	30 T1D, 30 controls, 2–5 yrs	Casecontrol	Yes	dmft, HbA1c	dmft ↑; HbA1c–caries +	Higher dmft in poor control	Glycemic influence on ECC
Thankappan (2024)	India	100 T1D, 100 controls, 2–6 yrs	Cross-sectional	Yes	dmft/deft	deft ↑ (NS)	Slightly higher deft	ECC prevalence relevance
Nasim (2022)	Pakistan	132 T1D, 132 controls, 7–12 yrs	Comparative	Yes	DMFT, xerostomia	DMFT ↑; xerostomia ↑	Higher DMFT, dry mouth	Salivary risk factors
Kamalova (2019)	Russia	7–15 yrs	Comparative	Yes	DMFT, GI	DMFT ↑; GI ↑	Higher DMFT, gingivitis	Periodontal relevance
de Oliveira (2018)	Brazil	46 T1D, 46 controls, 5–12 yrs	Cross-sectional	Yes	DMFT/dmft, saliva	dmft ↑; pH ↓	Higher dmft, lower pH	Salivary mechanisms
Iordanishvili (2017)	Russia	34 T1D, 25 controls, 5–7 yrs	Observational	Yes	dmft	dmft ↑*	Higher dmft; SD implausible	Narrative only
AlBadr (2021)	Saudi Arabia	69 T1D, 140 controls, 6–12 yrs	Cross-sectional	Yes	DMFT	DMFT ↑	Higher DMFT	Limited ECC relevance

The symbol ↑ denotes higher values in children with T1D compared to control subjects, while ↓ indicates lower values. The symbol = signifies no significant difference between the groups. An asterisk (*) marks statistically significant differences as reported in the original study, and NS indicates non-significant differences. Abbreviations: T1D: Type 1 diabetes; ECC: Early childhood caries; DMFT: Decayed, missing, and filled permanent teeth; dmft: Decayed, missing, and filled primary teeth; deft: Decayed, extracted, and filled primary teeth; HbA1c: Glycated hemoglobin; OR: Odds ratio; NS: Not statistically significant; GI: Gingival index.

### Risk of bias within studies

Risk-of-bias assessment using the JBI tools indicated that all 12 included studies fell within a low-to-moderate risk range, with none classified as high risk. Five studies met the majority of JBI criteria and were therefore rated as having moderate to low risk of bias, as detailed in Table 3 [19–22, 26]. Table 3 provides a detailed summary of the item-level JBI assessments for all included studies. The remaining seven studies were assessed as having a moderate risk of bias, primarily due to limited adjustments for confounders, insufficient reporting of examiner calibration, or inadequate detail regarding sampling procedures and sample-size justification [23–25, 27–30]. Only a few studies clearly reported strategies for addressing potential confounding variables, such as oral hygiene behaviors, socioeconomic factors, and dietary practices [19–21]. Despite these variations, the overall quality of the studies was sufficient to support a reliable narrative synthesis.

### Individual study results

The findings were heterogeneous across studies, reflecting variations in age ranges, diagnostic criteria, caries definitions, and assessment methods. However, several recurring patterns emerged across specific outcome domains.

### Dental caries in primary dentition

Nine studies reported caries experience in the primary dentition. Several studies indicated higher dmft scores in children with T1D, with statistically significant differences observed in some cases [26, 28, 31]. However, the study by Iordanishvili et al. reported unusually narrow standard deviations and a markedly larger between-group difference compared to the other included studies. These atypical data patterns suggested potential measurement or reporting issues, leading to cautious interpretation of its findings. Other studies found similar dmft values between children with T1D and healthy controls, with only minimal or clinically negligible differences [19, 20, 22]. Collectively, the available dmft data suggested only small between-group differences, with values showing limited variability and no consistent direction of effect. Even in studies where higher dmft was reported in the T1D group, the magnitude of difference was generally limited. Overall findings remain inconsistent, primarily due to heterogeneous age ranges, differences in dentition reporting, and variability in glycemic control. Evidence specifically for children aged ≤71 months is limited and insufficient for firm conclusions.

### Salivary characteristics

Six studies evaluated salivary parameters [19–21, 23, 26, 29]. A remarkably consistent trend of salivary hypofunction was observed among children with T1D, characterized by reduced unstimulated salivary flow, decreased buffering capacity, and more acidic salivary pH. These alterations collectively suggest impaired salivary protection and increased susceptibility to enamel demineralization and early caries development.

### Gingival and periodontal health

Five studies reported gingival or periodontal outcomes [23, 24, 26–28]. Across these studies, children with T1D frequently exhibited higher GI scores, elevated PI scores, and greater BOP. Notably, periodontal inflammation was often observed even when dmft scores did not differ between groups, suggesting a distinct biological pathway associated with metabolic dysregulation and inflammatory burden.

**Table 2:** Critical appraisal items from the JBI utilized for risk of bias assessment

**Question number**	**Appraisal question**
Q1	Q1: Were the criteria for inclusion in the sample clearly defined?
Q2	Q2: Were the study subjects and the setting described in detail?
Q3	Q3: Was the exposure measured in a valid and reliable way?
Q4	Q4: Were objective, standard criteria used for measurement of the condition?
Q5	Q5: Were confounding factors identified?
Q6	Q6: Were strategies to deal with confounding factors stated?
Q7	Q7: Were the outcomes measured in a valid and reliable way?
Q8	Q8: Was appropriate statistical analysis used?
Q9	Q9: Was the response rate adequate, and if not, was the low response rate managed appropriately?

Abbreviations: JBI: Joanna Briggs Institute; Q: Question.

**Table 3:** Risk of bias assessment (using JBI critical appraisal checklist)

**Study**	**Q1: Inclusion criteria**	**Q2: Study subjects**	**Q3: Exposure measured**	**Q4: Condition measured**	**Q5: Confounders identified**	**Q6: Strategies for confounders**	**Q7: Outcomes measured**	**Q8: Statistical analysis**	**Q9: Response rate adequacy**	**Comments**	**Overall judgment**
Blumer (2018)	Yes	Yes	Yes	Yes	Yes	Partial	Yes	Yes	Yes	Clear criteria and reporting	Low
Manjushree (2022)	Yes	Yes	Yes	Yes	Unclear	Partial	Yes	Yes	No	Limited confounder control	Moderate
Sheikh (2017)	Yes	Yes	Yes	Yes	Unclear	No	Yes	Yes	Unclear	Mixed-age sample	Moderate
Dubey (2018)	Yes	Yes	Yes	Yes	Unclear	No	Yes	Yes	Unclear	Unclear sampling method	Moderate
Lai (2017)	Yes	Yes	Yes	Yes	Yes	Yes	Yes	Yes	Yes	Strong methodology	Low
Salehi (2024)	Yes	Yes	Yes	Yes	Yes	Yes	Yes	Yes	Yes	Consistent dmft reporting	Low
Thankappan (2024)	Yes	Yes	Yes	Yes	Partial	Partial	Yes	Yes	Yes	Minor confounders	Low-Moderate
Nasim (2022)	Yes	Yes	Yes	Yes	Partial	Partial	Yes	Yes	Unclear	Heterogeneous sample	Moderate
Kamalova (2019)	Yes	Yes	Yes	Yes	Partial	No	Yes	Yes	Unclear	Inadequate confounder data	Moderate
de Oliveira (201)	Yes	Yes	Yes	Yes	Yes	Yes	Yes	Yes	Yes	High-quality reporting	Low
Iordanishvili (2017)	Yes	Yes	Yes	Yes	Partial	Partial	Yes	Yes	No	Implausible standard deviations	Moderate
AlBadr (2021)	Yes	Yes	Yes	Yes	Partial	Partial	Yes	Yes	Unclear	Some unclear domains	Moderate

Abbreviations: JBI: Joanna Briggs Institute; Q: Question; dmft: Decayed, missing, and filled primary teeth.

### Glycemic control (HbA1c)

Seven studies examined the relationship between HbA1c and oral health outcomes [19, 21, 23, 24, 26, 28, 29]. Findings commonly indicated that poor metabolic control (HbA1c ≥ 7.5%–9%) was frequently associated with higher caries burden, more severe gingival inflammation, increased mucosal lesions, and reduced salivary flow. With the exception of one study, which found no significant association between HbA1c and caries severity [20], these discrepancies may reflect differences in operational definitions of metabolic control, variations in dietary supervision, and the influence of fluoride exposure or oral hygiene practices.

### Preventive oral health behaviors

Four studies assessed preventive oral health behaviors [23, 27, 30, 32]. Common findings included underutilization of routine dental care, irregular tooth brushing, inconsistent fluoride use, and a lack of preventive service integration within T1D medical follow-up. A notable finding from Iordanishvili et al. demonstrated that quarterly supervised preventive dental visits over 12 months led to significant improvements in gingival health and oral hygiene [27]. This underscores the importance of integrating dental care into pediatric diabetes management models.

### Summary of results

Across the 12 studies, evidence for a definitive increase in ECC in young children with T1D remains inconclusive. However, consistently altered salivary dysfunction, periodontal inflammation, metabolic control indicators, and gaps in preventive care appear to collectively contribute to early oral vulnerability.

## Discussion

This systematic review synthesized current evidence on ECC and related oral health outcomes in children with T1D, with a particular focus on primary dentition (≤71 months). Although findings across the 12 included studies were heterogeneous, several consistent biological and behavioral patterns emerged. While evidence did not conclusively demonstrate a higher ECC burden in preschool-aged children with T1D, recurrent observations of salivary hypofunction, reduced buffering capacity, acidic salivary pH, and early gingival inflammation suggest potential oral vulnerability in this population.

### Interpretation of caries findings in the primary dentition

Caries outcomes in primary dentition varied across studies. Several investigations reported significantly higher dmft scores among children with T1D [26–28, 31, 32], while others observed comparable caries experiences between diabetic and non-diabetic groups [19, 20, 22]. One study documented lower dmfs scores among diabetic children relative to both healthy controls and those with phenylketonuria [33]. These inconsistent findings likely reflect differences in age ranges, diagnostic criteria, socioeconomic status, preventive behaviors, and glycemic profiles across studies. Importantly, only a limited number of studies reported primary dentition outcomes specifically for children aged ≤71 months, restricting firm conclusions within the ECC age window.

### Biological plausibility: Salivary and periodontal alterations

Despite variability in caries findings, strong biological signals consistently emerged across secondary outcomes. Salivary dysfunction—characterized by reduced unstimulated flow, diminished buffering capacity, and lower pH—was reported in multiple studies [19–21, 23, 26, 29]. These parameters are critical protective mechanisms against demineralization and bacterial acid challenge; thus, their impairment may increase long-term susceptibility to caries, even in the absence of immediate differences in dmft values. Similarly, gingival and periodontal inflammation was frequently observed among children with T1D [23, 24, 26, 27, 30]. Elevated GI, PI, and BOP values were common findings, suggesting heightened inflammatory responses potentially linked to metabolic dysregulation. Importantly, periodontal changes often appeared independent of caries severity, indicating distinct biological pathways influenced by systemic glycemic control, microbial composition, and immune responses.

### Influence of glycemic control on oral-health outcomes

Seven studies examined HbA1c in relation to oral health [19, 21, 23, 24, 26, 28, 29]. Poor glycemic control (HbA1c ≥ 7.5%–9%) was frequently associated with increased caries susceptibility, more pronounced gingival inflammation, reduced salivary flow, xerostomia, and mucosal lesions. These findings align with established physiological pathways through which chronic hyperglycemia contributes to oxidative stress, altered immune responses, and microvascular changes. However, two studies did not observe a significant association between HbA1c and caries outcomes [20, 33]. These discrepancies may reflect variations in dietary supervision, oral hygiene habits, fluoride exposure, or study design. Young children with T1D may also experience closer parental monitoring, which could mitigate caries risk despite suboptimal glycemic values.

### Preventive behaviors and healthcare utilization

Four studies assessed preventive oral health behaviors [23, 27, 30, 32]. A recurring pattern of underutilization of routine dental services was evident. Despite regular medical follow-up for T1D management, dental attendance was irregular, brushing frequency varied widely, and preventive fluoride use was inconsistent. The strongest evidence for the benefit of integrated care came from Iordanishvili et al., who demonstrated significant improvements in gingival inflammation and oral hygiene following quarterly supervised dental visits over a 12-month period [27]. This suggests that structured, multidisciplinary approaches could mitigate the biological vulnerabilities documented in children with T1D.

### Comparison with previous reviews

Previous systematic reviews examining oral health in children with T1D have generally reported a higher prevalence of periodontal inflammation, salivary changes, and, in some cases, increased caries experience. However, most of these reviews included mixed-age populations, often combining primary and permanent dentition or failing to isolate the ECC-specific age window. Consequently, their findings cannot be directly applied to ECC. For example, Rapone et al. [34] described a clear association between T1D and periodontal inflammation, as well as links between poor glycemic control and adverse oral health outcomes. However, the review did not stratify outcomes by dentition stage or preschool age, making it difficult to draw conclusions specific to ECC. Similarly, Banyai et al. [18] summarized broader oral health implications of pediatric diabetes and identified consistent inflammatory and salivary alterations, but did not provide dentition-specific or ECC-focused analysis. In contrast, the present review prioritized ECC criteria (≤71 months) and primary dentition outcomes; however, broader-age studies were retained when they provided extractable primary dentition data or relevant secondary oral health parameters to support biological interpretation. This methodological focus clarifies that current evidence does not confirm a higher ECC burden among preschool-aged children with T1D, despite biological indicators of vulnerability. Our findings align with earlier literature in demonstrating recurrent salivary dysfunction, lower buffering capacity, acidic pH, and early gingival inflammation, all of which suggest underlying susceptibility that may precede measurable caries differences in early childhood. Taken together, this review refines insights from earlier broad-age reviews by demonstrating that while periodontal and salivary markers are consistently altered in children with T1D, ECC itself remains inconclusive in the preschool years. This divergence between biological indicators and caries outcomes may reflect closer parental supervision, dietary regulation, and medical oversight in younger diabetic children—factors that may attenuate early caries risk even in the presence of metabolic vulnerability.

### Strengths and limitations

This review possesses several notable strengths, including the prioritization of ECC age criteria (≤71 months) and a focus on primary dentition, a comprehensive multi-database search strategy conducted in accordance with PRISMA 2020 guidelines and supported by prospective PROSPERO registration, and the use of standardized data extraction procedures alongside a formal JBI risk-of-bias assessment. Additionally, the review integrates both biological and behavioral oral health parameters, allowing for a broader understanding of the multifactorial influences on early oral health in children with T1D. Nevertheless, the findings must be interpreted in light of several limitations: only a small number of studies reported primary-dentition-specific data for children within the ECC age range; considerable heterogeneity existed in outcome definitions, diagnostic criteria, and measurement approaches; reporting of salivary, periodontal, and metabolic markers was inconsistent; and no longitudinal designs were available to clarify causal relationships over time. These constraints limit the strength of conclusions regarding true ECC differences but simultaneously underscore important gaps that warrant targeted future research.

### Implications for clinical practice

Although ECC severity did not differ consistently between groups, repeated observations of salivary impairment, gingival inflammation, and inadequate preventive dental engagement indicate that oral vulnerability may precede overt caries. Therefore, embedding oral health assessment, caries risk evaluation, and anticipatory guidance within pediatric diabetes management is both reasonable and potentially high-yield. Clinicians should emphasize early dental referral, consistent brushing with fluoride toothpaste, caregiver-supported oral hygiene, dietary counseling (particularly regarding free-sugar reduction), and regular preventive visits, especially for children with suboptimal glycemic control or salivary dysfunction.

### Implications for future research

Future studies should report caries outcomes separately for children ≤71 months, utilize standardized diagnostic criteria (dmft/ICDAS), and systematically measure salivary and periodontal parameters. Harmonized definitions of glycemic control, detailed behavioral assessments, and prospective longitudinal or interventional designs are urgently needed. Such approaches will help determine whether the biological and behavioral vulnerabilities observed in early childhood translate into a higher caries burden later in life.

## Conclusion

This systematic review provides the first ECC-focused overview of oral health outcomes in preschool-aged children with T1D, restricted to the primary dentition (≤71 months). Across the available evidence, no consistent increase in ECC was identified in young children with T1D compared with healthy peers. However, recurrent findings of reduced salivary flow, lower buffering capacity, acidic salivary pH, and early gingival inflammation reveal meaningful biological vulnerabilities that may precede observable caries differences. These alterations, combined with variable oral hygiene behaviors and underutilization of preventive dental services, underscore the need for proactive monitoring rather than reassurance based solely on dmft values. Given these patterns, early and routine integration of oral health assessment into pediatric diabetes care is warranted, including caries risk evaluation, anticipatory guidance for families, and structured preventive follow-up, particularly for children with suboptimal glycemic control or signs of salivary dysfunction. Evidence remains limited due to small sample sizes, heterogeneity in study designs, and inconsistent reporting of primary dentition outcomes. Future research should prioritize age-specific ECC reporting, standardized diagnostic methods, comprehensive measurement of salivary and periodontal parameters, and longitudinal designs capable of clarifying whether early biological susceptibilities translate into a higher caries burden later in childhood.

## Supplemental data

Supplemental data are available at the following link: https://www.bjbms.org/ojs/index.php/bjbms/article/view/13488/4128.

## Data Availability

All data supporting the findings of this study are available in the published articles included in the systematic review, which utilized narrative synthesis only; no meta-analysis was conducted. Additionally, no new datasets were generated or analyzed during this study.
